# Perspectives on relative energy deficiency in sport (RED-S): A qualitative case study of athletes, coaches and medical professionals from a super league netball club

**DOI:** 10.1371/journal.pone.0285040

**Published:** 2023-05-03

**Authors:** Justine O’Donnell, Chris White, Nick Dobbin

**Affiliations:** 1 Department of Health Professions, Manchester Metropolitan University, Manchester, United Kingdom; 2 Department of Health and Well-being, Wrexham Glyndwr University, Wrexham, United Kingdom; The University of Lahore, PAKISTAN

## Abstract

**Background:**

Research into relative energy deficiency in sport (RED-S) has increased substantially over recent years given the impact on athletes’ health and performance. Most studies have considered sports that place emphasis on the aesthetics, endurance, or weight-restriction. Fewer studies exist in team sports. Netball is a team sport yet to be explored despite players potentially being at risk of RED-S given the high training volumes, sporting culture, internal and external pressures, and small network of coaches and medical professionals. A qualitative case study was used to explore the perspective of athletes, coaches, and medical professionals on RED-S.

**Methods:**

Semi-structured interviews were conducted with 13 players, 4 coaches and 4 medical professionals affiliated to a Super League club. Interviews were recorded and transcribed *verbatim*. The data was analysed using thematic analysis.

**Results:**

Five main themes were identified in this study. Awareness of RED-S amongst athletes and coaches was generally inadequate whereas medical professionals had some awareness of RED-S. Some athletes used contraception to reduce discomfort/pain during menstruation whilst others expressed concerns around long-term contraceptive use and previous menstrual cycle disturbance. Sporting demands, individual and contextual factors, and a preoccupation with body image were associated with nutritional restriction, whilst appearance was a source of internal and external pressure. External pressures also extended to coaches, assessments/feedback, social media, and commentary. Strategies suggested to reduce the risk of RED-S included “hard hitting cases”, multidisciplinary team involvement, and support from the governing body.

**Conclusion:**

The findings of this study provide insight into factors potentially associated with the risk of RED-S from an athletes, coaches, and medical professional perspective. This insight can be used to increase overall awareness of RED-S in key stakeholders as well as improve the recognition for the pressures netball athletes face that might alter the level of risk.

## Introduction

Relative energy deficiency in sport (RED-S) is a multifaceted health syndrome described in 2014 by the International Olympic Committee [1], and encapsulates numerous physiological and performance-related changes that occur primarily due to low energy availability (LEA) [1–3]. RED-S encompasses the female athlete triad which is the most scientifically supported element of RED-S, and draws on relationship between LEA, menstrual dysfunction, and bone health [4]. RED-S reflects a deeper complexity that can involve athletes’ mental state and the cardiovascular, gastrointestinal, endocrine, reproductive, skeletal, renal and central nervous system [1, 4–6]. The potential adverse effect of RED-S from a health and performance perspective [1] has resulted in research exploring key stakeholders’ knowledge and awareness of RED-S (e.g., female cross-country athletes; [7]), athletes experiences of RED-S (e.g., endurance athletes; [5]), the physiological implications (e.g., endocrine function; [3]), and risk factors and symptoms [8, 9].

Despite the widespread interest in RED-S, awareness and knowledge of this syndrome is relatively poor amongst athletes and their support teams. This might have implications for the recognition of RED-S-specific policy and guidance documents, key risk factors and screening tools (e.g., RED-S CAT), signs and symptoms, and management approaches that are emerging. In several studies amongst athletes, including lightweight rowers [10], para-athletes [11] and cross-country runners [7], knowledge of this syndrome was generally low. Further, in a qualitative study by Langbein et al. [5], it was reported that greater knowledge and awareness of training demands, nutrition, disordered eating and having professional support could all aid in the recovery from RED-S. This latter point is important, as often in sport, athletes will seek support from coaches, peers, sport scientists/nutritionist, and medical personnel. However, within these groups, research suggests that knowledge and awareness is also limited [6, 10, 12, 13]. Kroshus et al. [12] reported that ~67% of athletic trainers working in the USA (*n* = 285) had not heard of RED-S, though a greater proportion had heard of, and recognised symptoms associated with, the female athlete triad. This finding is largely positive, particularly in the context of women’s sport, but might suggest that other populations such as male athletes could have symptoms associated with RED-S that go undetected. These results corroborated those of Tenforde et al. [13] and Gillbanks et al. [10] who reported that over half of health care providers (doctor, nurse practitioners, physical therapist, athletic trainer) had not heard of RED-S. In specific reference to coaches, fewer studies exist, but the findings from those that have explored this group suggest that knowledge of RED-S is limited, and that coaches are more concerned with performance outcomes than the long-term health of athletes [6, 14, 15]. Whilst the limited knowledge and awareness might be justifiable in many instances given RED-S is relatively new and many perceived this topic to fall outside of their scope of practice, there is little doubt that greater knowledge and awareness could be integral to minimising the risk of RED-S and support the recovery process [16]. To this end, further work is needed to explore the perspectives on RED-S across other athlete populations and support teams involved in yet unexplored sports.

Efforts have been made to extend research on RED-S and associated risk factors to other populations beyond female aesthetic and endurance athletes (e.g., recreational exercisers, male cyclists, synchronised swimming; and male combat athletes [17–20]), however little is available on team sport athletes. Current research using team sport athletes has explored risk factors and symptoms associated with RED-S or female athlete triad such as menstrual function, bone health and energy availability in female footballers [21–25] and volleyball players [26]. Numerous sports have received little attention despite potentially having an athlete groups at risk of the female athlete triad, and by extension RED-S. One such example is netball, a team sport that is characterised as a high-intensity intermittent sport, and that is growing in both popularity and professionalism. There are several reasons for this lack of focus at present including RED-S being a relatively newly defined syndrome, team-sport athletes not typically considered at high risk unlike those where the link between anthropometric measures and performance outcomes are more closely linked (e.g., gymnastics) [6]. Netball typically involves a small support team of coaches with limited or inconsistent support from nutritionists, sport scientists and medical personnel. This, combined with the demands of high-level sport, the culture (e.g., moving from an amateur to professional culture; [27]) and organisational framework [28] in which players, coaches and other support staff operate, netball players may be at risk of symptoms associated with RED-S.

Overall, the current evidence suggests that RED-S can have a significant impact on athletes’ health and performance, and that some athletes are potentially at greater or lesser risk due to their knowledge of this syndrome and that of their support team. In sports such as netball where there is a changing culture, high training and match demands, and a small coaching/medical network around athletes, it is important to explore current awareness of RED-S, potential risk factors, and strategies to moderate risk. Therefore, as a first step to exploring RED-S in netball, this study sought to use a qualitative approach to explore the perspectives of players, coaches, and medical professionals on RED-S within netball.

## Methods

### Study design

A qualitative case study design was used, focusing on a specific group of elite athletes, coaches, and medical professionals, a typically hard to access group who were likely to have unique perspectives on the topic. The overarching focus was concerned with participants’ experiences through gaining insight into how they interpreted their social reality [29]. This meant that qualitative research was most suited as it offers an opportunity to gain in-depth insight into people’s ideas, feeling and perceptions [30]. It is important to note that these goals for qualitative research mean that there are clear differences between qualitative and quantitative research with regards to recruitment, sampling, and data analysis. Structured representative samples in quantitative research allow for statistical generalisation to the wider population from which samples are drawn [31]. Meanwhile, qualitative research is focused on providing “illumination and understanding” of complex issues, rather than conclusions that are immediately generalisable to a wide population [32]. The generalisation of qualitative research comes through the in-depth insight gathered, and findings from this study are likely to be important and insightful for a broader category of cases (e.g., female team sport players).

### Procedures

A semi-structured, face-to-face interview was conducted to allow the researcher to respond to the participant’s non-verbal cues and ‘body language’ as well as using unscripted probes to elicit novel and potentially significant insight on areas that emerged during the interview [33, 34]. Probes were developed to ensure they were not leading and generally included questions such as “why do you think this”, “do you think others feel the same”, “can you give me an example of” as well as probing for context around “when”, “where” and “who was involved”. Separate interview schedules were developed for players, coaches, and medical personnel. Participants were initially asked if they were aware of RED-S. If not, a definition and list of symptoms was provided to enable the interview to continue. All interviews were scheduled to take ~60 minutes.

### Participants & sampling strategy

Participants were selected in accordance with purposive sampling, directed by a criterion sampling strategy [31]. Purposive sampling allowed for a selection of participants who were affiliated with one professional netball club in the United Kingdom. This sample was therefore deliberately non-random as participants were selected on the basis that they would be well-positioned to provide responses directly relevant to the research aims [31]. Participants included coaches who had a minimum of one season at a club, HCPC registered physiotherapists, GMC registered doctors, and netball players aged 14 years and over (Table 1).

**Table 1:** Participant characteristics and key contextual information.

Pseudonym	Role	Playing squad	Age Group	Total weekly hours training/competing across all sports	Additional participation in sport/exercise outside of the club’s schedule.
Annie	Player	Academy	14–17	15.5	Lacrosse, netball, tennis, rounders, hockey, spin cycle
Lara	Player	Academy	14–17	15.0	Netball, hockey, swimming, running
Lilly	Player	Academy	14–17	9.5	Netball
Leah	Player	Academy	14–17	18.0	Netball, lacrosse, hockey, tennis, athletics
Liberty	Player	Academy	14–17	12.0	Netball, handball, volleyball
Maddie	Player	Academy	14–17	13.0	Hockey, athletics
Danielle	Player	Academy	14–17	20.5	Netball, hockey, athletics, tennis, golf
Bo	Player	Academy	14–17	13.0	Lacrosse, netball, hockey
Lola	Player	Super League + Int	Open	12.0	None
Charlie	Player	Super League	Open	10.0	Gaelic football
Kelly	Player	Super League	Open	12.0	None
Emma	Player	Super League + Int	Open	14.0	Netball
Amelia	Player	Super League + Int	Open	8.0	None
	Role	Squad	Years at the club	Additional experience at previous clubs or in other sports	Main responsibilities
Luna	Coach	U15/U16/U17	1	Netball coach	All aspects of netball training.
Dillon	Coach	Super League	5	None	On-court fitness coach
Tilly	Coach	Super League	1	Squad, head, and international netball coach	Talent ID, strategic management
Gemma	Coach	Super League	5	Netball coach, netball head of performance	Defence coach
Nadia	Doctor	Super League	1	Olympics, international and national swimming, Premiership football, triathlon, marathon, and tennis	General medical cover for squad and matchday support
Dominic	Doctor	Super League	1	Olympics, international and national swimming, Premiership football, triathlon, marathon, and tennis	General medical cover for squad and matchday support
Lizzie	Physiotherapist	Super League	3	Olympics, Commonwealth Games, Welsh rugby union	Assessment and rehabilitation
Rita	Physiotherapist	Super League	2	Women’s football, GB Taekwondo, GB Judo.	Match-day physiotherapy

### Ethical considerations and trustworthiness

Ethics approval was granted for the study by the Faculty of Health and Education at Manchester Metropolitan University (Application No. 34474). Each interviewee, and where applicable, the caregiver, received an information sheet. Prior to the interview, all participants signed an assent/consent form, and for those under 18 years, a caregiver consent form was also signed. Consideration for maintaining confidentiality and anonymity of participants was paramount, especially since our sample contained, national- and international-level athletes and coaches. As a result of this, all participants will be referred to using pseudonyms with the club remaining anonymous.

The exclusion criteria meant that those who have or had previously been diagnosed with an eating disorder were excluded. However, sensitive material related to the discussion of menarche, menstrual cycles and the use of contraception were included as they were recognised as highly relevant to the study. Therefore, as there remained a risk of the interviewee’s recollections causing upsetting reminders, a post-interview support leaflet was given along with relevant contact details of support services.

To assure the quality and credibility of the case study, the research required conscientiousness, consistency, and trustworthiness. There was also a need to balance degrees of involvement and detachment between the research team and the topics under scrutiny [35]. The researchers recognised early in the study that were was a need to take steps towards detachment in key stages of the process, especially as the lead author was heavily involved in the world that we were researching. To support, this the final author, who was not involved in the interviewing process or familiar with the club used, but is familiar with the topic, acted as a ‘critical friend’ and independently reviewed the interview schedules and transcripts. This allowed for a degree of neutrality and impassivity [36]. That said, we do recognise a degree of involvement was present as, for example, the first author had a personal interest in this topic and was loosely associated with the club used in this case study. This involvement was essential in the process of gaining ‘insider knowledge’ on sensitive topics (e.g., menstrual function, contraceptive use, disordered eating) from athletes, coaches, and medical professionals working in elite sport. Therefore, whilst involvement was ever present, we strived for an adequate level of detachment throughout the research process, bringing about more reality-congruent conclusions.

### Data analysis

All interviews were transcribed *verbatim* and then read by the first and last authors for accuracy. The transcripts was then imported into NVivo (Version 12, QSR International Pty Ltd. 2018). The qualitative data was analysed using a process of coding, data categorisation, and thematic representation that attends to description and analysis [37]. The focus on description and analysis from the thematic representation reflects a mixed deductive and inductive approach where some initial topics were discussed (e.g., knowledge, symptoms, risk factors etc.) based on the research aims and existing literature, and others emerged from the open-ended questions that were not previously considered [37]. Initially, the first and last authors independently read through transcripts and developed a list of initial codes that aligned to the topics of interest. The final list of codes was reorganised by both the first and last author together to form categories that became single larger areas of interest relevant to the topic. Thematic representation of the data was the final process with a focus on description and analysis, as discussed by Roulston [37]. Description allowed us to address aspects such as ‘what the participants know’, whilst also allowing them to build on the discussion to provide insight into essential features, the context or parties involved as well as inform evaluative questions posed by the researchers [37].

## Results and discussion

In total, 13 players, 4 coaches and 4 medical professionals agreed to participate, with key descriptive information on each participant presented in Table 1.

The results of this study can be categorised into four overarching themes, awareness of RED-S and associated symptoms, menstrual function and use of contraceptives, risk factors, and strategies that related to reducing the risk of RED-S.

### Awareness of RED-S and associated symptoms

In accordance with previous research [7, 10–12, 38], there was an overall lack of awareness of RED-S with only one player having heard of this syndrome. Some of the players interviewed were able to associate RED-S with “fuelling”, “energy intake and efficiency”, “dietary needs” and “how much energy a person is burning” (Amelia, Lola, Gemma), but couldn’t discuss the syndrome further or provide a definition. A similar level of knowledge was observed for all the coaches, with none being familiar with the term RED-S or having been formally introduced to the syndrome, but were able to identify some symptoms and even recognise players that may have displayed associated symptoms in the past (see Table 2). Coaches appeared to have indirect markers that would give them insight into athlete’s current wellness that can be loosely associated with RED-S or LEA. For example, one of the coaches described how she thought that the coach’s eye was “really important to see the sort of behaviours and how an athlete presents when they come into training, so if an athlete is falling over a lot and it’s not something they generally do that’s a concern for me, sunken eyes is a concern, bags under their eyes you know, little physical exertion”. The strength and conditioning coach also referred to how “adapting to the training programme” gave him insight, and if players were not adapting as expected “that is a bit of a red flag”.

**Table 2:** Coaches & medical personnel RED-S awareness and management approach.

Participant	Awareness or knowledge of RED-S*	How did you hear about RED-S (if applicable)*	Symptoms perceived to relate to RED-S from participants	Identified an athlete with RED-S symptoms?	Aware of RED-S CAT tool	Who should be responsible for RED-S screening/diagnosis?
Tilly (coach)	No	-	Change of behaviours, sunken eyes, bags under eyes, fatigue, tiredness	Yes	-	Stated manager, nutritionist (acknowledged role of MDT meetings).
Luna (coach)	No	-	Changes in eating behaviour	No	-	MDT
Gemma (coach)	No	-	Fatigue, training withdrawal (unable to maintain intensity and engage in sessions)	No	-	MDT
Dillion (coach)	No	-	Fatigue, not getting stronger/gaining weight on training programme	Yes	-	MDT
Nadia (doctor)	Yes	Sports Medicine Masters	Restrictive diet, (vegetarians and vegans), age (adolescents in particular), body habitus (tall and thin)	No	No	MDT
Dominic (doctor)	Yes	Sports Medicine Masters	Fluctuation in weights, changes to menstrual cycle, injury rate, stress fractures.	No	No	MDT
Lizzie (physio)	No	-	Poor sleep patterns, poor energy levels, poor performance, anxiety	No	No	MDT
Rita (physio)	Yes	Podcast	Repeat fractures, frail, tired all the time, menstrual dysfunction.	No	No	MDT

Note: * Asked and answered before participants were provided with a written definition of RED-S. CAT = RED-S clinical assessment tool. MDT = multi-disciplinary team (player, coach, medical personnel, nutrition etc.).

The overall level of knowledge and awareness for all players and coaches are encapsulated by Amelia and Dillion, respectively:

*Amelia: “No ‘I’ve never heard of RED-S, but I know about energy balance, and if you have too little, you won’t play at your best. I learnt that from my social media; it comes up quite a bit. Also, we have a nutritionist at England and regular sessions with a nutritionist at [club name]”*.

*Dillion: “I didn’t even know what the abbreviation was, I didn’t have a huge understanding of it, my nutrition knowledge is basic but having read it now, I would suggest that that it is a common issue in a lot of sport especially female sport and netball specifically.”*

These results suggest that there is some awareness in this netball club, but that further research, promotion, and education on factors related to RED-S is needed if we want key stakeholders to recognise the risk factors and correctly identify symptoms before health and performance is significantly impacted.

The data from the interviews suggested that social media appeared to be a key source of information pertaining to nutrition and energy balance such that this was mentioned *before* nutritionists at a national and club level. This is common in today’s world, particular with female athletes who are three times more likely to use social media for information on nutrition than males, as it provides rich, quick, and easy-to-access information [39]. Gaining insight on RED-S from the nutritionist was noted by some senior players, and it was assumed by coaches that players with the national team would have greater awareness that young players. This is reflected in the following quote:

*“A lot of the girls who have come through the England pathway would have had various talks and education sessions through the years, so I’d say a lot of your older players would have more of an idea than your younger girls” (Gemma)*

However, our results do not support this despite nutritionists and medical personal being closely situated within the athlete’s ‘sportsnet’ at national level. Therefore, it should not be assumed by club-level coaches that key topics such as RED-S will be covered elsewhere, and that there is a responsibility of clubs to provide knowledge and awareness where possible through appropriate personnel (i.e., doctors, physiotherapists, nutritionists, dieticians).

After being presented with a definition and list of symptoms, it was suggested that player do display some of these symptoms throughout a season, which is an important findings in this study. Whilst some of the reasons behind coaches linking the symptoms to netball will be discussed further below, it is anticipated that the training and match demands, culture of the sport changing from amateur to professional, and other contextual factors such as travel, sponsor demands, and clothing, amongst others [27], impose a demand on players that might increase the risk of RED-S. Indeed, one coach referred to the display of symptoms being a “culmination of a lot of things”, and the head coach would “try and pick up trends” in objective and subjective data such as “jumps” and “RPEs” through a “Performance Data Management System” (Tilly). The same coach also described how they would:

*“expose these athlete to scenarios…and rehearsal camps [in which] the nutritionist’s work fed into with the strength and conditioning coach, and I was managing on-court sessions, particularly when they were in those physical states which seem associated with RED-S” (Tilly)*.

These findings are reassuring and suggest that there is consideration (e.g., increased perceived effort for the same given workload) for factors associated RED-S that are put into practice such as “rehearsal camps” to ensure there is a multidisciplinary team approach when athletes demonstrate signs of overtraining, overreaching, or even burnout. This is evident despite limited knowledge or awareness of RED-S specifically.

All but one of the medical professionals demonstrated a good level of awareness of the female athlete triad and RED-S, and were the only group to link RED-S to menstrual cycle disturbances, fatigue, and risk of bone-related injury. In part, this greater awareness was the result of further education that covered topics such as “the female athlete triad” (Dominic) which remains highly relevant, and is a core feature of RED-S [40]. Two medical personnel highlighted that they learnt additional symptoms after reading the definition sheet and that they were unaware of “what to do about do about it and how to pick it up” (Nadia). The overall level of awareness amongst this groups is summarised by one of the physiotherapists who stated:

*“Yes, in the physio world it [RED-S] is starting to be talked about a little bit more. I think I knew maybe the signs as in decreased menstrual cycles, risk of fractures and to look out for signs that maybe they weren’t fuelling properly. But I think there are a lot more symptoms that before reading this [the definition sheet] I wasn’t aware of” [Rita]*.

### Menstrual function and use of contraceptives

Athletes’ responses to questions about their menstrual cycle, impact of menstruation on sport performance, and use of contraception are presented in Table 3. Most (54%) athletes reported having a regular menstrual cycle; however, 3 did report a loss of menses for at least three months and one athlete for over 18 months. Six (46%) athletes reported that menses had or does have a negative impact on netball performance, and most of those using OCP/IUD did so for sporting reasons. The choice of OCP/IUD was best summarised by Lola, Kelly and Charlie who noted that having a period was “an extra thing that makes me feel uncomfortable and don’t to worry about it” and that using contraceptive “controls this really well”, “was a nice side effect” and was “definitely handy because if you’ve got a game on your first day then it’s not a good feeling”. These findings largely concur with Clarke et al. [41] who explored contraceptive use across various football codes in Australia. Clarke et al. [41] reported that 38% of athletes used contraceptive to control their menstrual cycle and 36% use it to reduce menstrual pain. Furthermore, five of the participants in this study also reported heavy menstrual bleeding which has been reported as another reason for OCP/IUD use (~ 22%; Clarke et al. [41]). In the younger participants, only one was using OCP/IUD with this being related to acne control.

**Table 3:** Athletes’ responses to menstrual cycle, impact on sport and contraception usage.

Athlete (group)	Age of menarche (years)	Regular menstruation (menstrual flow)	Menses had/has a negative impacts on netball performance	Loss of menses for ≥ 3 consecutive months	vb usage	Reasons for OCP/IUD usage
Annie (U15)	Not commenced	-	-	-	-	-
Lara (U15)	Not commenced	-	-	-	-	-
Lily (U15)	11	Yes (heavy)	Yes	No	No	-
Leah (U16)	13	N/A (regular)	No	No	Yes, currently	Skin
Liberty (U16)	13	Yes (regular)	No	No	No	-
Maddie (U16)	13	Yes (regular)	No	No	No	-
Danielle (U17)	12	Yes (heavy)	Yes	No	No	-
Bo (U17)	13	Yes (regular)	No	No	No	-
Lola (Super League)	13	N/A (heavy)	Yes	Yes	Yes, currently	Sport
Charlie (Super League)	12	N/A (heavy)	Yes	No	Yes, currently	Sport
Kelly (Super League)	13	No (regular)	Yes	Yes	Yes, previously	Sport
Emma (Super League)	16	No (heavy)	Yes	Yes	Yes, previously	Sport
Amelia (Super League)	13	N/A (regular)	No	No	Yes, currently	Skin

Note: OCP: oral contraceptive pill; IUD: intrauterine device. Menstrual flow is subjective and not based on recognised criteria for menorrhagia.

Whilst our data suggest that 6 athletes were using OCP/IUD, it is also important to understand the reasons for no longer using contraception. In Clarke et al.’s study [41], they noted that the main reasons for disuse was negative side effects (31%; mood swings, weight gain, depression/anxiety), wanting a natural approach (17%), and it no longer being needed (15%). In this study, athletes discussed how they were no longer using an OCP/IUD:

*“to come off it was a personal reason and not totally necessary to stay on it. It was more of a do I really need to be taking something else that can affect hormone balance and obviously all that kind of thing” [Kelly]*

Another athlete provided similar sentiments, linking this to their bone health and long-term menstrual function:

*“I’d say about a year and a half ago I stopped the contraceptive. I was on the injections, so I stopped the injections because obviously it is not good for your bone health. So, I didn’t want to get performance issues and the doctor said I should come off it. I was just like I am not going on anything I do not want anything to affect my periods in that way down the line, I do not want anything to be broken” [Emma]*

These results suggest that, at an elite level, the change in sport culture, a greater focus on winning and the influence from coaches and medical personnel, provides a source of pressure to consider and use OCP/IUD. Indeed, several players referred to the pressure they felt to conform to an expected standard. For example, one athlete shared their experiences at an international level:

*“we had a talk from the doctor about the pill and if you were a player who really struggled during major competition times with your period, taking the pill could be a good way to control that. There are some girls at England who really, really struggle with their period they can’t go to training because their period is that bad and, in that case, I’d say yes, it’s a good option. There’s quite a lot of different cultural views and cos its contraception people don’t want to take it” (Amelia)*.

Whilst there is an acknowledgement of the potential benefits of using a contraceptive from a sporting perspective, there was also a dilemma presented that athletes deal with around following advice from those in netball (e.g., coaches, medical personnel) and the perceived performance benefits against the potential long-term implication on health and menstrual function.

### Risk factors associated with an increase in risk of RED-S

Throughout the interviews, numerous risk factors emerged that are associated with RED-S. These included nutritional restrictions, the sporting demands and culture, internal and external pressures, and anthropometric assessment.

### It was common for players to experience/practice nutritional restriction caused by the sporting demands, individual factors, and preoccupation with body image

A key theme that emerged from the data was intentionally or unintentionally restricting eating, which was most common in the Super League players. Academy players eating behaviours were generally good because of the support from caregivers. For example, Liberty noted how she “has probably focused more on needing to eat healthier and my Dad and Mum are big on me eating healthy and good food, so that probably started to be more important as I played”. Similarly, Annie was supported by her Mum, “my Mum is a physiotherapist, so she kind of knows a lot about the nutritional stuff, so she helps to know what to eat before training [and] what the benefits are like from different foods and how long they last”. Whilst restrictive eating patterns in the younger athletes was not overly evident in this study, these findings suggest that the support network around younger individuals seem to be important as a source of information and responsibility for their nutritional intake. However, this support network must place the short- and long-term health and wellbeing of the athlete at the centre of all decisions concerning aspects related to RED-S, especially in younger athletes [42]. Therefore, the autonomy athletes have over their eating patterns should be a key focus, with particular attention paid to vulnerable athletes who may not receive nutritional support from caregivers and maybe more susceptible to the influences of external sources of information (e.g., social media).

When concerning the senior players, dieting and the intentions behind this became a key focus of discussions with restricted eating patterns being common [43]. Whilst these factors are important to consider, the restricted eating patterns were often unintentional and sometimes unavoidable. Several participants recalled how it was difficult to consume sufficient nutrition intake during their time in education due to a busy schedule. They also discussed how their inability to meal plan, consume food during travel to and from games, cook, and afford sufficient and healthy food often results in restrictive eating behaviours. In part, this was summarised by Lola who recalled that:

*“during university I know I didn’t eat enough like it was bad to be honest with you. I know a lot of people have terrible eating habits in university, but I’d say probably because I was really busy, and I was doing a lot of travel [for netball]… I wasn’t massively skilled in terms of cooking different food, so I just ended up not really eating much food to be honest with you. Looking back at it, it horrifies me, I don’t even know how I made it through”*.

Some of the difficulties that were discussed by players were acknowledged by the coaches and medical personnel, again, particularly in reference to the senior players who are “in fulltime work and then go to training sessions which are late in the evening, 8–10 o’clock” (Dominic). Dominic went on to say that:

*“You [players] leave for work at 7.30–8am and get back at 11pm so certainly it puts stress on the body… nutrition, food and their eating habits are very difficult. It’s hard to eat adequality and properly and eat the right things when you’re away from home for such a prolonged period …so it certainly will impact”*.

Therefore, like other amateur or semi-professional sports, the recognition of limited finances, understanding of optimal sport nutrition, and time management should encourage clubs to implement sessions focused on improving the athlete’s nutritional knowledge, cooking skills, and planning.

Another key area that emerged to help understand periods of restricted eating concerned remarks from coaches or other key stakeholders about body image, which worked to reassure this behaviour and encourage players to continue. Such findings have been observed in elite athletes across various sporting groups, where in Bentley et al.’s [44] study, it was noted how coaches would often comment on how “fit”, “good” and “athletic” athletes looked, and how this reflected their judgment of overall sporting performance and players conformability to the ‘professional athlete identity’ [44]. In this study, several participants noted how they would receive compliments about how they look despite acknowledging that the behaviours to achieve this were “not ok”. This is reflected in Lola’s responses:

*“because I did actually lose quite a lot of weight [referring to a period of dieting], so I always have like a weird feeling about that because I know that my eating was not ok and I wasn’t eating enough food. I lost a lot of weight got a lot of compliments for it obviously, and also, I did feel my game kind of excelled during that time”*

These complimentary remarks made by others regarding weight loss appeared to conflict with the perceived implications of rapid weight loss on injury risk by some players. For example, Amelia noted a link between rapid weight loss and injury in other players previously:

*“There is a huge injury risk because they just drop muscle mass and weight, and then they have not got support structures [a]round their limbs, so then they do an ACL that year or a really bad ankle roll. I’ve seen so many have a major injury after they have dropped the weight, which I think it’s funny isn’t it, they say you’re going to be a better player if you lose all that weight, but it has to be done properly, very controlled, and you have to the take the mental aspect into it”*.

This is an important finding which may be underpinned by the relationship between LEA and musculoskeletal injury both of which form key elements of RED-S [1]. Therefore, as the quote above suggests, it’s important to consider the implications of comments around body shape, mass (weight) and composition within this group of athletes. Whilst the meaning behind these comments was likely meant in positive terms, there appeared to be less reflection on how athletes had reached the desired body shape, mass (weight) or composition.

### Preoccupation with appearance acts as a source of internal and external pressure on netballers

It was evident throughout all interviews that there was an understanding netballers are required to be physically, technically, tactically, and mentally able to cope with the demands of training and competition. It was also clear that anthropometric characteristics severed multiple purposes within the sport (e.g., recruitment, selection, training focus), resulting in a preoccupation with body size, shape and mass (weight). In trying to understand the role preoccupation with appearance has when considering risk factors, it became apparent that pressure came from internal and external sources.

### Internal pressures

Considering the internal pressures that were referred to by almost all players, the data suggested that academy player had an impression that they must conform to the *ideal* body shape/composition that enabled them to be “strong, thin, athletic, dynamic, defined and a certain weight” [Danielle, Liberty and Lily]. Similar sentiments were echoed by the senior players, with many stating that “there is a look that is usually very tall, slim people” and that “in the past, people have wanted me to look a certain way and be a certain weight”. Such statements have been noted in other athlete groups, particularly female athletes. For example, Bentley et al. [44] noted how many elite-level athletes felt there was a need to have an *ideal* body for their sport which seemed to be based on comparisons with teammates. Interestingly, this *ideal* body was rarely defined precisely. Nonetheless, the notion of gauging an *ideal* body from others seems to potentially place academy players at a slightly higher risk; that is, they need to look like those in the senior team by any means possible given this is considered by many an important part of being an elite athlete. One participant discussed how this might add pressure for younger athletes:

*“I think maybe the younger players coming through might be more susceptible [to RED-S]. They’ve got to step into the limelight, (team name) are quite good at bringing through the youngsters…It’s that pressure of sort of coming on and you know some of them train really well to nail down their place, the pressure of new playing environments and competition where the grade of the match is even higher… so if they’re feeling under more pressure that can potentially have an impact in terms of their diet even from an anxiety perspective or if I lose weight or do this, do that, it’s going to make me perform better” [Lizzie]*

Interestingly, this quote links losing weight to enhancing performance, which as we discussed earlier, is not aways the case when considering the mediating/moderating injury risk. Such findings have been observed previously amongst a group girls and young women involved in an array of sports where they were balancing what they term a ‘performance culture’ focused on physical characteristics needed for sporting excellence and an ‘appearance culture’ that was emphasised outside of the sport (e.g., at work, at school, on social media) [45]. In netball, often this ‘appearance culture’ was brought on-court [46]. For example, Lola stated:

*“I do think there’s a big pressure, personally I’m not naturally a skinny minnie [and] as a netballer especially because we wear little dresses to play*. *I think there’s a kind of pressure there that people might not think about unless they’re not super skinny”*

### External pressures

External pressures were identified by almost all athletes interviewed, and included the influence of selection by coaches based on anthropometric characteristics, feedback from coaches on performance and assessments, commentators’ remarks, and social media.

Both senior players and coaches discussed how subjective and objective data concerning body composition, mass (weight) and shape were used within the selection decisions around a player and reinforced as important by coaches. Arguably this could be positive for some athletes as this provides a form of reward for efforts around nutrition and training [44]. However, for others, it is likely that anthropometric measures are used *against* athletes to allow for a degree of questioning players’ conformability to the expected standards within elite sport and may result in inappropriate and dangerous behaviours. For example, Emma described an instance where focus on anthropometric measures caused distress that impacted her over a prolonged period:

*“My coach deselected me from a tour because she said I was too big. So originally, I was in the 12 selected to be in the game so she put me back a step and the reason for that she [the coach] told me I was too big. So then when I went home, I was like how can I lose weight in the two weeks I had off so I literally ate nothing. That is a massive mental barrier for me. I was getting on the court thinking I was too fat to play and couldn’t do this. That obviously affected my play for at least a few years”. [Emma]*

There was a degree of understanding around the risk of anthropometric measures or vocalised perceptions by coaches/medical staff, largely by reflecting on their own experience as a player or relating it to other sports. One coach discussed how she once experienced a coach using selection as a method to place emphasis on weight loss. Several coaches also discussed how measures or perceptions of a player’s anthropometry profile, when vocalised, can be mis-interpreted by players with direct implications that associate with LEA and RED-S. This was summarised in the following quote:

*“yeah I think so because especially younger players may think it effects their selection. It may be a comment from a coach in the past, you need to be lighter, you need to be heavier so suddenly their right ok we are being weighed now, right I’m putting on weight so you have to be really, really careful.” [Dillion]*

The impact of selection to represent the national team also appeared to act as an external source of pressure with dedicated guidance on nutrition given, requirements around body composition, and completion of screening/assessments. For some athletes, this greater guidance and input from sport science/coaches might facilitate performance and allow players to demonstrate the ‘professional athlete identity’ expected as this levels as has been reported previously [44]. However, for other, involvement at this level might increase the risk of LEA due the additional training volumes, and the emotional and mental pressure [6]. Furthermore, the increased discussion and focus on nutrition and body composition might result in some undesirable behaviours as this is likely to be interpreted as an important component by athletes [6]. These points emerged from the data across multiple participants, and are summarised by:

*“…it is such a shame… a lot of people at England, that I see, nutritionally are not eating enough at all they are following diets like vegetarian and not getting enough protein, and when they’re at camp, they’re not learning to increase eating enough and a lot of it is mental pressure cos they’ve been told to lose weight and it’s about control of what you’re eating”. [Amelia]*

And

*“there’s a lot of pressure, the England girls want to do really well and stay in the England squad. “When there is competition for places there is this psychological merry-go-round they might think if I lose weight, if I do this or will I perform better if I skip meals”. [Lizzie]*

### Feedback from coaches on performance and assessments

The collection, interpretation and feedback of objective data is commonplace in sport and considered essential by some within the ‘elite’ environment to track athletic development, inform selection, and monitor athletes [44]. In exploring this topic, players and coaches provided insight into their experience of various forms of assessments, though we highlight that it was minimal at the club used and there were no requirements for players to share data with others. The participants expressed an unwillingness to share their data. For example, Emma noted that she “would never openly share it [data on weight or body fat] with coaches, I would share it with one teammate because I know she will support me in whatever”. This suggests that players accepted these measurements were often taken but they were given control over who to share their data with. However, it is possible this type data was shared with coaching and medical team at international level or other clubs as is often the case in elite sport.

Coaches understanding of the implications assessments of anthropometry could have on athletes was evident and seemed to have emerged from their own experience. A negative experience involving the assessment of anthropometry that was reflected by all coaches was described by Tilly:

*“So negative experiences*, *so they basically were fat testing*. *They called it fat testing rather than skinfolds…they weighed us every week and reported it to the coach… I’ve had it that if I don’t lose weight; I won’t get selected*. *“Honestly when I think about the abuse that I’ve had*, *it’s been awful”*.

However, despite the coaches terming their own experience as “abuse” and “awful”, some players did suggest that coaches have applied pressure on players to meet a profile they deem to be suitable for that level of competition and playing position. For example, one senior player said:

*“yes I think I have had a lot of pressure… some coaches have really tried to make me lose weight and not been very nice about it… so then I’ve been upset about it and tried to change drastically which obviously wasn’t nice and then I quickly realised it isn’t sustainable to eat lettuce leaves for about a month”. [Emma]*

What also emerged mid-way through the interviews with coaches was a realisation that the player profile they desired had evolved substantially since their playing years. They described the playing position in relation to anthropometric measures (e.g., “centre count generally below 7.5% [body fat]”) and clothing sizes (“centre court players are more size 8”), but then acknowledged that players are now required to be “lean” and “athletic” [Rita, Tilly, Amelia]. As such, the description of what an elite netball should look like seemed to be an ‘old-school’ view of the *ideal* body without consideration for the changing demands and culture of the game. Interestingly, whilst all coaches had a view on the *ideal* body for a netballers, it appears that past ‘scars’ had meant that coaches largely off-loaded the responsibility of assessing and supporting athletes, referencing the need for a multidisciplinary team, specifically a nutritionists. One example of why the coaches felt they “need to bring in other specialist practitioners to support the process” was based on the following scenario:

*“I remember saying to an athlete once, which I learnt as a coach not to say again, I think we need to work a lot more on your speed and looking at power to weight ratio. She [the athlete] replied ‘Why don’t you just say I am fat’, and I thought I don’t need to address that, I thought she looked slow, and I needed to say to S&C and the nutritionist, I think we need to look at this; it’s really difficult to have those conversations. Have I had these conversations, of course, I’ve sat down loads with [Emma] in a performance review, but it just doesn’t seem to come across well. I think you get more retaliation from athletes thinking if you deselect them it’s about their weight not lack of performance.”*

Several studies have explored the role of commentators or other media personnel within areas associated with a susceptibility to RED-S such as body image, body mass (weight) and general aesthetics. For example, Kerr et al. [47] reported how one parent of a young gymnast questioned why television commentators announced the weight of athletes during their broadcasting. Further, Marfell [46] noted how commentators, or the wider media, often reinforce a feminized representation of players through focus on their looks, relationships, sexual orientation, and lives outside of the game (e.g., media work, familiar life). Whilst some aspects noted here were not explored in this study, some players, and in particular Emma, recalled that:

*“I think it’s about how you look. I think for some teams, they [the coaches] would rather not have someone like me in my position. So, this season I am playing goal attack, so even on commentary they are saying she is not the most agile goal attack we’ve ever seen, she’s quite slow blah blah blah”*

Such insights agree with those previously mentioned in netball, and suggest that a focus on appearance and how some anthropometric factors are perceived to affect performance (e.g., agility) are recognised by players as an external pressure that could lead to behaviours associated with LEA, the female athlete triad, and RED-S.

Another external pressure centred around social media and the impact this might have on eating behaviours that subsequently increase the risk of LEA and symptoms associated with the female athlete triad and RED-S. Several academy players did acknowledge that social media, such as Facebook and Instagram, can play a role, stating that “social media has a massive impact on how we look at ourselves and our bodies” [Lily] and “sometimes social media can be like quite damaging”. It was, also a concern noted by Super League players (e.g., Amelia) and medical personnel (e.g., Dominic), where it was noted that:

*“On a lot of social media, they [the athletes] are very low percent body fat so it looks good you can see definition but that’s not ideal for training you need a bit of body fat along with the muscle for training” [Amelia]*.

This apparent external pressure that causes athletes to focus on body image is a concern and worthy of further research in netball given the link between disordered eating, body image and perfectionism when understanding risk factors of RED-S [2].

### Strategies discussed to mitigate the risk of RED-S in netball

Over the last two years, there has been a slow but necessary transition in the research towards interventions that can support athletes at risk of RED-S or those diagnosed [18, 48, 49]. In this study, the most mentioned strategy to reduce risk of RED-S at [club name] was education, of which informal talks, use of case studies, and athlete/coach/medical discussions were most prominent. Many participants believed pre-season was the optimal time to deliver education on RED-S with shorter top-up sessions offered throughout the season and post-season. Dillon suggested the following approach:

*“offer quite hard-hitting case studies and real-life examples of how it can potentially lead to issues, or injuries, things with performance because a coach is always going to want to get an extra 2–3% performance and have player availability so you sell it in that way.”*

During these education sessions, athletes expressed a need to discuss the health implications of LEA and how this was associated with the female athlete triad and RED-S. Participants also expressed a need for the athlete to understand the risks and benefits of contraception and cultural implications. Finally, athletes noted that specific and tailored nutritional advice could be helpful. Indeed, Kelly noted how “some athletes need to know more about working out calories and macro(nutrient) breakdown”. This suggests a more individualised approach might be required when implementing or research interventions for athletes at risk.

All coaches and medical personnel believed screening for risk factors associated with LEA and RED-S was necessary, and that an MDT approach would be optimal to effectively managing this with in the athletes. None of the medical personnel had seen the RED-S CAT before but did indicate that it could be a useful screening tool when described by the interviewer.

Governing bodies were perceived as key stakeholders in addressing topics like LEA, the female athlete triad and RED-S, and are able to improve the awareness of these topics in netball. Their access to greater resources, ability to fund further research into RED-S, and collate extensive data on netball players was deemed highly significant by coaches, medical personnel and some senior players. One of the coaches reflected on the involvement of the governing body on topics like RED-S stating: “England netball yes, they have full time personnel, they have all the data, they have a huge responsibility, do they take that responsibility on, no?”. Whilst this suggest the governing body could be well-position, it also indicated that coaches were not aware of any initiative or approach on the topic of RED-S at the time of the interview. Although the evidence suggests there is need to act as soon as possible, there are questions that remains around how current strategies (e.g., education) should be approached in-light of the current level of knowledge, the internal and external pressures of players, and how athletes perceive other contextual factors to influence their behaviour altering the risk of symptoms of the female athlete triad and RED-S.

### Strength and limitations

Use of a qualitative approach is a strength of this study, whereby we have been able to provide rich detail in the form of quotations from elite-level coaches, medical personnel, and athletes at junior through to Super League and international level. Whilst statistical generalisability is not possible nor appropriate, the supporting material around our participants own words may resonate with others. Use of three groups provided novel insight on occasions of agreement or shared thoughts (e.g., assessments) and incorrect perceptions (e.g., athletes on England pathways). The use of a case study design may be a potential limitation as our findings may only reflect the culture and procedures at one club as well as those athletes who were willing to discuss this topic. We recommend that future research continues to consider team sport athletes in discussions on RED-S as well as explore the efficacy of screening tools and interventions to reduce risk.

## Conclusions

This study aimed to explore the awareness, risk factors, and strategies associated with RED-S amongst players, coaches, and medical professionals at a single Super League netball club. The findings of this study contribute a first insight into factors surrounding RED-S within an elite-level netball environments whilst also synthesising data from athletes across the age spectrum, coaches and medical professionals. The findings of this study suggest that awareness of RED-S amongst netball athletes and coaches was inadequate, and that there are several risk factors that might increase the risk of RED-S. It appeared that these risk factors differed between the groups, and had a bigger influence on senior players likely due to the pressures and change in culture at this level. Doctors and physiotherapists might be well-placed to support athletes as awareness and recognition of symptoms was good, but diagnosis, management and treatment options are areas medical personnel can improve. In this club, assessment strategies used was minimal, but conversations about body weight appear to be a high-risk approach when delivered by non-specialists. Our results offer some strategies as recognised by players, coaches and medical personnel that future work might consider when supporting netball players maintain an appropriate balance between health and performance.
